# Comparison of CyTOF assays across sites: Results of a six-center pilot study

**DOI:** 10.1016/j.jim.2017.11.008

**Published:** 2018-02

**Authors:** Michael D. Leipold, Gerlinde Obermoser, Craig Fenwick, Katja Kleinstuber, Narges Rashidi, John P. McNevin, Allison N. Nau, Lisa E. Wagar, Virginie Rozot, Mark M. Davis, Stephen DeRosa, Giuseppe Pantaleo, Thomas J. Scriba, Bruce D. Walker, Lars R. Olsen, Holden T. Maecker

**Affiliations:** a Human Immune Monitoring Center, Stanford University, Stanford, CA, USA; b Centre Hospitalier Universitaire Vaudois, Lausanne, Switzerland; c Ragon Institute of MGH, MIT, and Harvard, Cambridge, MA, USA; d Fred Hutchinson Cancer Research Center, Seattle, WA, USA; e Institute for Transplantation and Immunity, Stanford University, Stanford, CA, USA; f South African Tuberculosis Vaccine Initiative, Institute of Infectious Diseases and Molecular Medicine, Division of Immunology, Department of Pathology, University of Cape Town, Cape Town, South Africa; g DTU Bioinformatics, Technical University of Denmark, Copenhagen, Denmark

**Keywords:** CyTOF, Mass cytometry, Multicenter, Normalization, Standardization, Clustering

## Abstract

For more than five years, high-dimensional mass cytometry has been employed to study immunology. However, these studies have typically been performed in one laboratory on one or few instruments.

We present the results of a six-center study using healthy control human peripheral blood mononuclear cells (PBMCs) and commercially available reagents to test the intra-site and inter-site variation of mass cytometers and operators. We used prestained controls generated by the primary center as a reference to compare against samples stained at each individual center. Data were analyzed at the primary center, including investigating the effects of two normalization methods.

All six sites performed similarly, with CVs for both Frequency of Parent and median signal intensity (MSI) values < 30%. Increased background was seen when using the premixed antibody cocktail aliquots at each site, suggesting that cocktails are best made fresh. Both normalization methods tested performed adequately for normalizing MSI values between centers. Clustering algorithms revealed slight differences between the prestained and the sites-stained samples, due mostly to the increased background of a few antibodies. Therefore, we believe that multicenter mass cytometry assays are feasible.

## Highlights

• A multicenter mass cytometry study was performed among six international centers.
• A premixed antibody cocktail resulted in higher background for a few antibodies.
• Manual gating and clustering algorithms demonstrate that all sites were adequately consistent in performance.

## 1 Introduction

For a few decades, fluorescent flow cytometry has been the standard method for phenotypic analysis of mixed populations of cells. However, spectral overlap between fluorophores limits the number of simultaneous probes per experiment. Mass cytometry is an alternate method which uses elemental tags instead of fluorophores for labeling probes, allowing analysis by inductively-coupled plasma mass spectrometry. This typically avoids the issue of overlap between detection channels, and allows the routine use of > 40 simultaneous probes.

While challenging to coordinate, multicenter studies have been successfully performed in fluorescence flow cytometry (Finak et al., 2016, Kalina et al., 2015, Maecker et al., 2005). Although the number of research publications involving mass cytometry have continued to increase since the first publication of comprehensive immunological data generated by CyTOF by Bendall et al. in 2011 (Bendall et al., 2011), most studies have been performed in a single lab at a single site (Brodin et al., 2015) Few studies have tested machine-to-machine variation, and in recent years, the use of normalization beads to account for intra-instrument day-to-day variation has become common practice (Tricot et al., 2015, Finck et al., 2013). While one multicenter study was reported at a conference (Nasaar et al., 2015) and another multicohort analysis was recently published (Blazkova et al., 2017), no systematic multicenter CyTOF assessment has been published to compare issues of staining and instrument performance consistency. Here, we designed a staining panel from commercially-available reagents to allow measurement of major cell populations in cryopreserved PBMCs. We adapted a method (Sumatoh et al., 2017) to cryopreserve prestained cells to use as an external control for each of six centers across three countries. Reagents, unstained cells, calibration beads, and prestained cells were shipped to each center. Each center then performed the staining protocol on the unstained cells and then ran the newly-stained samples and the pre-stained samples on CyTOF2 instruments after daily tuning. The resulting files were sent back to the main center for standardized analysis by a single researcher.

## 2 Materials and methods

### 2.1 Materials and reagents

The human Peripheral Blood panel kit (#201304), containing 17 antibodies, MAXPAR Cell Staining Buffer (CSB), and MAXPAR Fix/Perm Buffer was purchased from Fluidigm Corporation (South San Francisco, CA). Additionally, CD56-Yb176 (#3176008B), CD7-Eu153 (#3153014B), CD33-Gd158 (#3158001B), Cisplatin (natural-abundance, 5 mM stock; #201064), Ir-intercalator stock solution (#201192B), and EQ four-element beads (#201078) were also purchased from Fluidigm. RPMI (HyClone #SH30027.01), FBS (heat-inactivated before use) (Atlanta Biologicals, #S11150), Pen-Strep-Glutamine (100 ×) (HyClone #SV30082.01), and Benzonase (25 × 10^5^ U/mL; Pierce Antibodies #88701), were purchased from Fisher Scientific. PBS (10 × stock; Rockland #MB-008), 0.1 μm spin filters (Millipore #UFC30VV00), and 5 mL blue-cap cell strainer filter tubes (Falcon #352235) were purchased from Fisher Scientific. DMSO was purchased from Sigma (#D8418). MilliQ water was used to make 1 × PBS (CyPBS). Note: it is important that all MilliQ water and buffers are stored in bottles that have not been washed with soap, due to the barium content of many commercial soaps.

Peripheral blood mononuclear cells (PBMCs) were isolated by Ficoll-paque density gradient centrifugation from a healthy donor leukocyte reduction system chamber obtained from the Stanford Blood Center. After isolation, the cells were dispensed into aliquots of 10 million cells per vial and cryopreserved in the vapor phase of liquid nitrogen until use.

### 2.2 Shipping of samples

The Human Immune Monitoring Center (HIMC) was the primary center and prepared all reagents for all six centers. For consistency, all reagents were purchased by the HIMC and then distributed to all sites. For each site, two packages were prepared. The concentrated antibody cocktail (see below), CSB, Fix/Perm Buffer, and EQ beads were shipped at 4 °C with gel cold packs. The aliquots of prestained samples (see below), unstained cells, Ir intercalator, and cisplatin were shipped on dry ice.

### 2.3 Preparation of antibody cocktail

The 20 undiluted antibodies were mixed at appropriate titer to create 368 μL of concentrated cocktail (Table S1), sufficient for both the prestained cell samples and for the site-stained samples. The cocktail was then split into aliquots of 6 × 35 μL for each site and stored at 4 °C, with the rest used on the day of mixing to create the prestained control cells.

### 2.4 Multicenter cell staining procedure

The staining protocol was modified from the manufacturer's “MaxPar Cell Surface Staining Protocol”. Cryopreserved PBMCs were thawed at 37 °C in a water bath, washed by resuspension in 10 mL of warm RPMI containing 10% heat-inactivated FBS, 1 × Pen-Strep-Glutamine, and Benzonase (25 U/mL final concentration). The cells were collected by centrifugation (RCF = 480, 10 min, room temperature), the supernatant removed, and the cells resuspended in fresh RPMI and centrifuged again. The cells were diluted to 1 mL in CSB, then measured for viability and cell count by Vicell (Beckman Coulter). Two aliquots (sample A and sample B) of 2 million viable cells each were dispensed into 5 mL polystyrene tubes (Falcon). 2 mL of CSB was added to each tube, centrifuged (10 min, RCF = 480, R.T.), and supernatant discarded by aspiration.

For antibody staining, the cells were resuspended in a volume of 50 μL CSB. Sixty-five microliters of CSB was then added to the 35 μL of concentrated antibody cocktail (100 μL total). The cocktail was placed in a 0.1 μm spin filter and centrifuged in a microcentrifuge (10 min, RCF = 14,000, R.T.) to elute. If necessary, the volume was adjusted to 100 μL with CSB. Fifty microliters of diluted antibody cocktail were then added to each cell sample, mixed by pipetting, and incubated at room temperature for 30 min. The samples were washed twice with 2 mL 1 × CyPBS (diluted to 1 × with MilliQ water), centrifuged (10 min, RCF = 480, R.T.) and supernatant removed by aspiration.

For cisplatin live-dead staining, 1.5 mL of 1 × CyPBS was added to a tube containing 2 μL of 5 mM cisplatin. A two-fold dilution was made by adding 750 μL of the cisplatin to 750 μL of 1 × CyPBS. The cells were resuspended in the residual volume of CyPBS, then 100 μL of diluted cisplatin were added to each sample and incubated for 5 min at room temperature (Note: do not exceed 5 min!).

Following the cisplatin incubation, the samples were washed twice with 2 mL CSB by centrifugation (10 min, RCF = 480, R.T.) and aspiration. The cells were resuspended in the residual volume by gently pipetting after the final wash/aspiration. Next, 2 μL of Ir intercalator stock were diluted with 2 mL of Fix/Perm Buffer and 1 mL added to each tube before overnight incubation at 4 °C.

The following day, the cells were each washed twice with 2 mL of CSB by centrifugation (10 min, RCF = 800, 4 °C) and aspiration. Each replicate tube (A and B) of cells was resuspended in 2 mL of CSB, then split into two tubes of 1 million cells (sample A1 and A2, sample B1 and B2). Samples A2 and B2 were then placed at 4 °C overnight.

The prestained control cells were processed as 7 aliquots of 2 million cells each, using the same protocol. During the final CSB resuspension, the aliquots were pooled, then re-split into 13 aliquots of approximately 1 million cells. Twelve aliquots were centrifuged (10 min, RCF = 800, 4 °C) and the supernatant removed by aspiration. Each cell pellet was resuspended in 100 μL of freezing media (10% DMSO in 90% heat-inactivated FBS) and frozen at − 80 °C until use (Sumatoh et al., 2017). The thirteenth aliquot was not placed in freezing media; instead, it was washed with MilliQ water and run on the CyTOF2 as a never-frozen reference (see below).

### 2.5 Sample acquisition on CyTOF2

Of the six sites, Centers 1, 4, 5, and 6 had CyTOF2 instruments (Fluidigm). Centers 2 and 3 had CyTOF1 (Fluidigm) instruments that had been upgraded by the manufacturer to CyTOF2 hardware specifications and software, resulting in machines comparable to CyTOF2 instruments (often called CyTOF1.5).

Prestained sample 1 was thawed on ice and transferred to a 5 mL polystyrene tube. Sample A1, A2, and prestained sample 1 were washed twice with 2 mL MilliQ water by centrifugation (10 min, RCF = 800, 4 °C), and aspiration. EQ calibration beads were diluted 10-fold in MilliQ water. The CyTOF2 was started and left to warm up for 20 min, then tuned with Tuning Solution (Fluidigm) according to the manufacturer's recommendation. From the QC log from the Results tab after tuning, the values for Resolution; Dual Slope values for Cs and Tm; Mean Dual Count Tb value; RSD (Dual) values for Tb, Cs, La, Tm, Ir; Mean 155Gd Dual counts; Analog Controls: Detector Voltage, Nebulizer Gas, Makeup Gas, and Current were recorded (Table S2).

Immediately before injection, each cell pellet was diluted to 1 mL (~ 0.8 × 10^6^ cells/mL) with the 0.1 × EQ beads and filtered into 5 mL blue cell strainer cap tubes. Each sample was acquired in 2 × 500 μL injections (using both sample loops) as a single FCS3.0 file. At this point, the raw FCS files were not normalized and EQ beads were not removed. The next day, sample A2, sample B2, and prestained sample 2 were processed and recorded in the same way.

### 2.6 Data analysis

The raw FCS files and QC log reports were sent back to Stanford University for standardized analysis. M. Leipold performed all manual gating analysis (Mac FlowJo X version 10.0.7r2; Supp. Fig. S6). In addition to the raw files, the FCS files were normalized in two ways: the Fluidigm normalizer built into the CyTOF2 v.6.0.626 software using the Passport P13H2302 (“Fluidigm v1”) and Passport P13H2302_ver2 (“Fluidigm v2”) (User Guide UG13-02_150501); and the MATLAB normalizer from Finck et al. (v.7.14.0.739 run in MATLAB R2012a; Finck et al., 2013). These batch-normalized files were then re-analyzed in FlowJo. All 151 FCS files are available at Flow Repository under accession **FR-FCM-ZY3Z**. Frequency of Parent and Median intensity data for all files were then exported for analysis in JMP (SAS) by G. Obermoser.

The two current normalization programs differ in their methodology: the Fluidigm algorithm included in the CyTOF software normalizes files according to a set of external reference values determined by Fluidigm (User Guide UG13-02_150501). This allows each file to be processed separately if desired, but does not allow for variation in peak mass sensitivity between different machines as observed in Tricot et al. (Tricot et al., 2015). Additionally, there is minimum required number of EQ beads per file in order for the normalizer to function properly (typically at least 50 beads per 100 s of data).

In contrast, the MATLAB algorithm uses the EQ beads within each file to create a local set of values per file (Finck et al., 2013). Next, all the files included in that batch are compared to generate a global calibration value, which is then used to normalize all the files together for each channel. This method allows for variation in peak mass sensitivities during the local value generation, but typically requires that all files to be compared are processed in the same batch (although a recent software update allows the designation of a reference file to which all others are normalized). This method does not have bead-count requirements to function, but normalization will be more representative of the file if an adequate number of beads are present.

### 2.7 Clustering analysis

Normalization is critical before any clustering analysis. Here, the MATLAB-all normalized files were chosen in order to have all 6 normalized files from each of the 6 sites (Table 1). In order for any advanced data analysis methods to be performed, the files must have the same parameter names. Therefore, due to parameter naming differences between some sites (see Discussion below), the file parameters were harmonized using the cytofCore package (https://github.com/nolanlab/cytofCore), using Center 2′s P1 sample as the reference template. After this, the files were gated in FlowJo X to Live Intact Singlet CD66 − CD45 + CD235ab − CD61 − events and re-exported as new FCS files. These new files were used for SPADE3 and for Cytofkit analysis (below), and have been uploaded to the FlowRepository record as a combined ZIPPED file.

**Table 1:** Summary statistics for each center, for number of files before and after normalization.

	Raw	Fluidigm v1	Fluidigm v2	MATLAB-pass Fluidigm	MATLAB-all
Number of files					
Center 1	6	4	4	4	6
Center 2	6	4	5	4	6
Center 3	6	0	0	0	6
Center 4	6	6	6	6	6
Center 5	6	6	6	6	6
Center 6	6	6	6	6	6
Total	36	26	27	26	36

Average CV - Frequency of Parent - all files all populations					
Center 1	11.5	11.0	11.1	11.6	14.3
Center 2	9.1	10.2	11.3	12.4	9.8
Center 3	12.8	–	–	–	16.2
Center 4	11.5	11.7	9.7	10.8	11.6
Center 5	13.9	14.1	15.1	14.9	30.0
Center 6	13.2	15.5	14.8	16.2	13.9
Average of all centers	12.0	12.5	12.4	13.2	15.9

Average CV - MSI - all files all populations					
Center 1	36.0	31.6	34.4	31.6	35.4
Center 2	16.6	19.7	25.6	21.5	17.2
Center 3	25.3	–	–	–	27.9
Center 4	32.5	31.1	34.8	30.1	30.4
Center 5	29.4	30.1	31.5	29.5	35.0
Center 6	29.9	39.8	30.0	27.8	26.7
Average of all centers	28.3	30.5	31.3	28.1	28.8

The clustering algorithms chosen were PCA, SPADE3 (pre-compiled Mac MATLAB stand-alone version; Qiu, 2017; http://pengqiu.gatech.edu/software/SPADE/) and Cytofkit (Chen et al., 2016; https://github.com/JinmiaoChenLab/cytofkit), which is an R software package containing components for PCA and tSNE outputs for ClusterX (Chen et al., 2016), Phenograph (Levine et al., 2015), and FlowSOM (Van Gassen et al., 2015) algorithms. To explore the effects of prestained vs site-stained samples, the files were run as three sets with each algorithm: the 12 P files only, the 24 AB files only, and all 36 ABP files. The set-up parameters for each algorithm can be seen as screenshots in Suppl. Fig. S8. In all cases, the 14 markers used for clustering were CD27-Er167, CD3-Er170, CD123-Eu151, CD33-Gd158, CD14-Gd160, CD19-Nd142, CD4-Nd145, CD8a-Nd146, CD16-Nd148, CD20-Sm147, CD11c-Tb159, CD45RA-Tm169, HLA-DR-Yb174, and CD56-Yb176. All output results for SPADE3 and for Cytofkit have been uploaded to the FlowRepository record as ZIPPED files.

## 3 Results

### 3.1 Creating and storing prestained samples

**Fig. S1:**
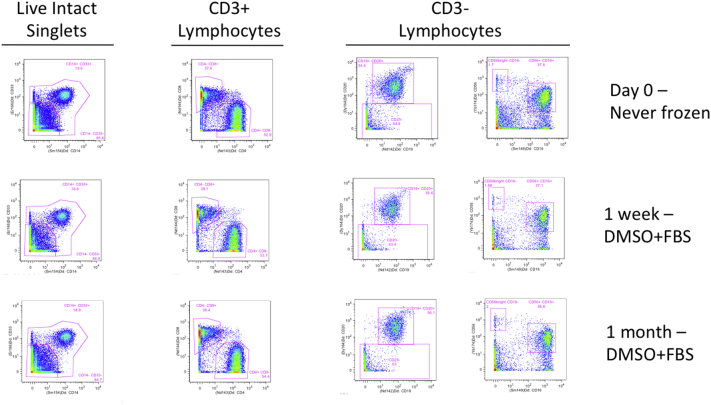
Effects of freezing and storage time on cell population Frequency of Parent. Cells were stained, then distributed into aliquots. One sample was run immediately as Never Frozen, while the remaining aliquots were resuspended in 10% DMSO in FBS and frozen at − 80 °C for varying lengths of time.

Prestained cell samples are commonly used for longitudinal standardization. However, until recently, there was no information on long-term storage of prestained samples for mass cytometry. In Nov 2014, a Cytoforum (http://cytoforum.stanford.edu) post mentioned rescuing an experiment by freezing in 10% DMSO/90% heat-inactivated FBS at − 80 °C when a CyTOF machine unexpectedly required repairs. This protocol was recently published in detail (Sumatoh et al., 2017). Therefore, after completing the entire staining procedure and including the post-Ir washes, we tested 10% DMSO/90% heat-inactivated FBS. We tested two timepoints: one week and one month of − 80 °C storage. These samples were compared with the results from another aliquot of the same cell staining experiment that had remained in CSB, had never been frozen, and had been run on the CyTOF2 as soon as staining was complete (never stored). At one week, the 10% FBS samples were unchanged relative to the never-frozen control (Fig. S1). There was no further change in frequencies in the one month sample. Repeating the freezing experiment gave similar results (not shown); therefore, 10% DMSO/90% heat-inactivated FBS was chosen as the storage media for the Prestained samples.

### 3.2 Comparisons to be made

**Fig. 1:**
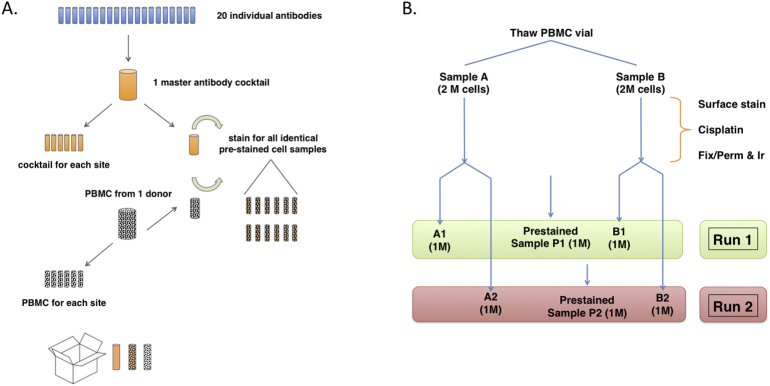
Scheme of multicenter experiment. A. Outline of reagent preparation for each site. A master cocktail was mixed from 20 undiluted antibodies. Six aliquots were made for distribution, then the remaining cocktail was used to stain PBMCs to make Prestained sample aliquots for each site. An unstained PBMC aliquot from the same donor was also shipped for staining on-site. B. Protocol for staining cells and sample acquisition. One PBMC aliquot was thawed and split, then stained on Day 1. On Day 2, each sample was split into two parts. Part 1 was run on the CyTOF that day, while part 2 was run on Day 3. Additionally, aliquots of Prestained sample were thawed and run each day.

There were two main purposes of the Multicenter Study (Fig. 1): first, to measure the reproducibility of each site performing their own cell staining. This was measured by comparing the A and B samples vs the Prestained samples. Second, running the same samples on two successive days tested the day-to-day variability of each instrument. To minimize variation due to antibody conjugation efficiency, only pre-conjugated antibodies commercially available from Fluidigm were used. To minimize variation in making the cocktail mixture, a master stock of concentrated antibody was mixed and undiluted aliquots were made for each site (Fig. 1). This same stock was also used fresh on the same day to make the Prestained samples. To minimize variation in cell frequency due to biological variation between donors, cryopreserved PBMC from a single donor was used to make the Prestained samples and additional PBMC aliquots were shipped to each site.

### 3.3 Effect of antibody cocktail storage on staining

**Fig. S2:**
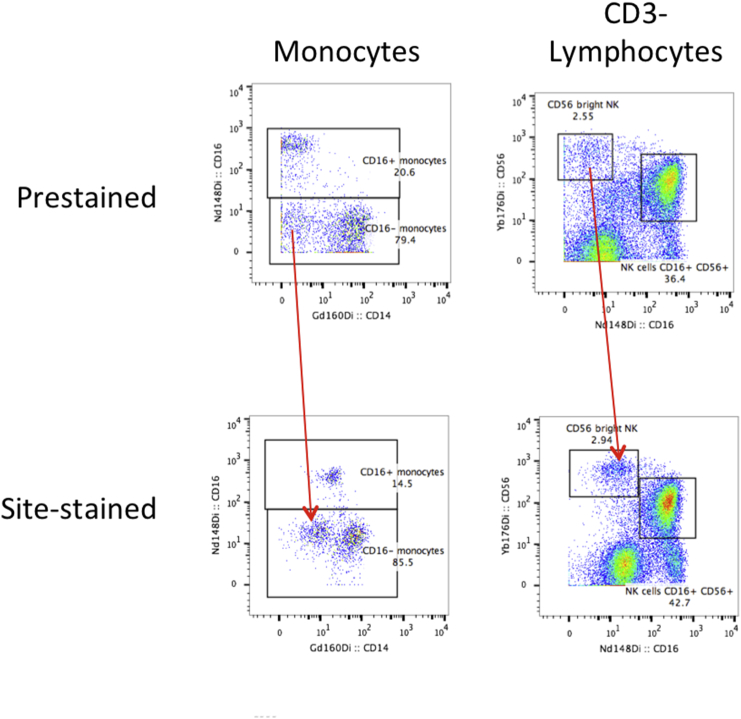
Effects of antibody cocktail storage. Aliquots of concentrated antibody cocktail were shipped to each site for on-site staining of PBMCs. Compared to the Prestained samples, higher background was seen for some antibodies in the site-stained samples.

Relative to the Prestained samples, site-stained samples from all six centers had much higher background for the negative population in several channels (Supp. Fig. S2). This does not appear to be a shipping artifact, as the two on-site Stanford centers were affected as strongly as the other four sites. It should be noted that not every antibody was affected: for example, the backgrounds for CD14 and CD16 were high, while those for CD3 and CD56 were unchanged. This was presumably due to antibody aggregation during storage as a concentrated cocktail, since separate aliquots of exactly the same cocktail preparation had been used fresh to process the Prestained samples. Due to delays in shipping and/or sample processing at each center, there was a minimum of two weeks between creation of the concentrated cocktail and the first site use for staining. Even with increased background, it was still always possible to resolve the populations of interest. The signal intensities of the positive populations were unchanged.

### 3.4 Normalization

The inclusion of the EQ calibration beads allowed us to batch-normalize all the raw files. The EQ beads contain four elements (Ce, Eu, Ho, and Lu) with a total of seven isotopes distributed across the CyTOF mass window. Therefore, using the beads as a constant reference throughout each sample allows normalization of the files by interpolation.

### 3.5 Intra- and inter-site differences-manual gating

**Fig. 2:**
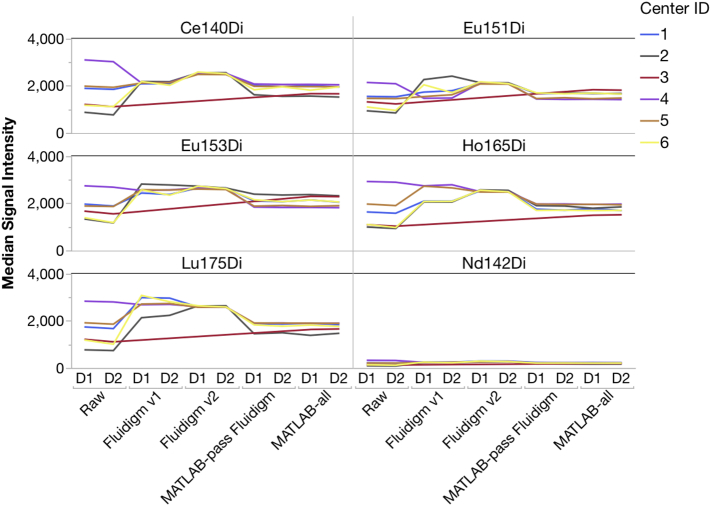
Signal intensities of the metal isotopes present in Fluidigm EQ Four-Element Beads vary by center. Each of the normalization methods removes some of the inter-center variation.

Variation in sensitivity between sites was observed even in the EQ calibration bead channels (Fig. 2). Each site was consistent from Day 1 to Day 2, indicating that each site's instrument was stable, but different from the other sites in sensitivity. Normalizing using either method made the six sites noticeably more similar in Median signal intensities.

**Fig. 3:**
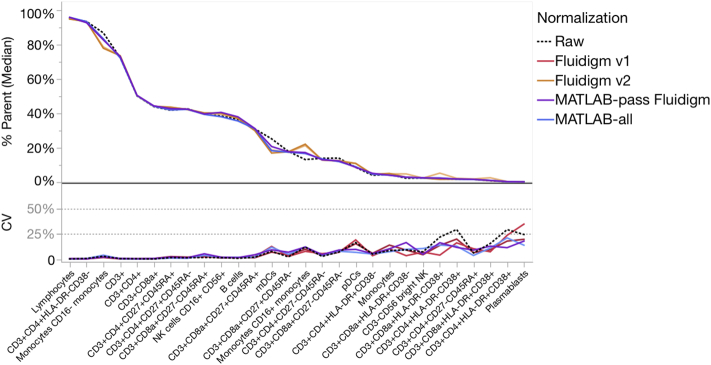
Effects of normalization on Frequency of Parent. The Frequency of Parent Median and CV were calculated for various manually gated cell populations for all samples, all centers combined. The effect of normalization was compared to the Raw data.

**Fig. S3:**
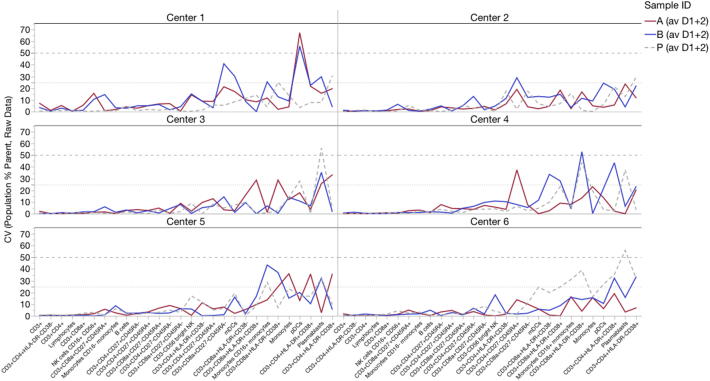
Center-by-center CV for Frequency of Parent of various manually-gated cell populations. Each line depicts the average of a separate pair of samples (A1 + A2, B1 + B2, P1 + P2). Raw, unnormalized data are depicted. As Frequency of Parent decreases, CV typically increases.

For all samples of all centers pooled together, there was little difference in Frequency of Parent between the raw files pooled from all sites and the same files normalized by either method (Fig. 3; Table 1; average CV < 20%; individual gated population CV typically < 25% in Table S3). This was expected, since all gates for each file were manually adjusted during analysis. Additionally, the CV of Frequency of Parent between sites was quite narrow, ranging only 12–16% (Table 1 average pooled CV < 16%). Even for low-frequency populations, the CV for individual centers seldom went above 30% (Supp. Fig. S3).

**Fig. 4:**
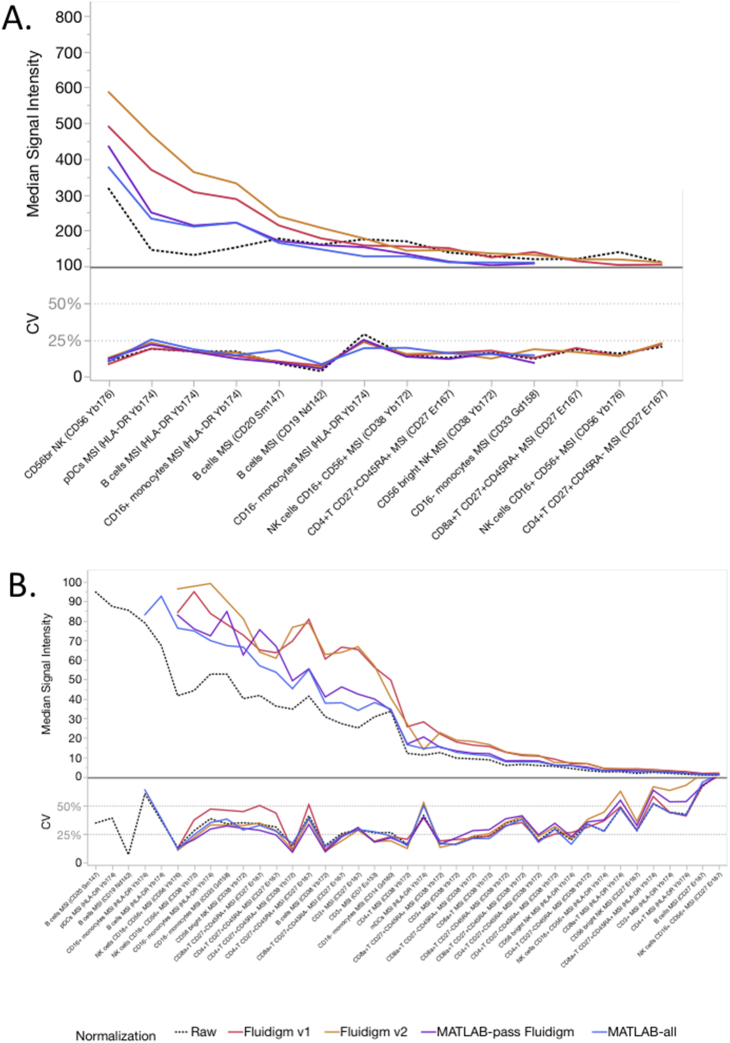
Effects of normalization on median signal intensity (MSI). The MSI median and CV were calculated for various manually gated cell populations for all samples, all centers combined. The effect of normalization was compared to the raw data. A. MSI ranging from 800 to 100 Dual Counts. B. MSI ranging below 100 Dual Counts.

**Fig. S4:**
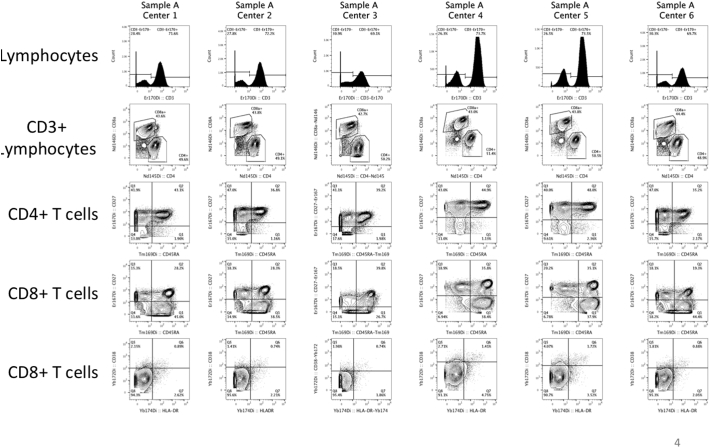
Sample A1 as an example of inter-center variation for on-site staining. Unstained cells were incubated with the concentrated antibody cocktail aliquot. Raw, unnormalized data are depicted. While variations in signal intensity between centers can be seen, all populations remain resolved.

**Fig. S5:**
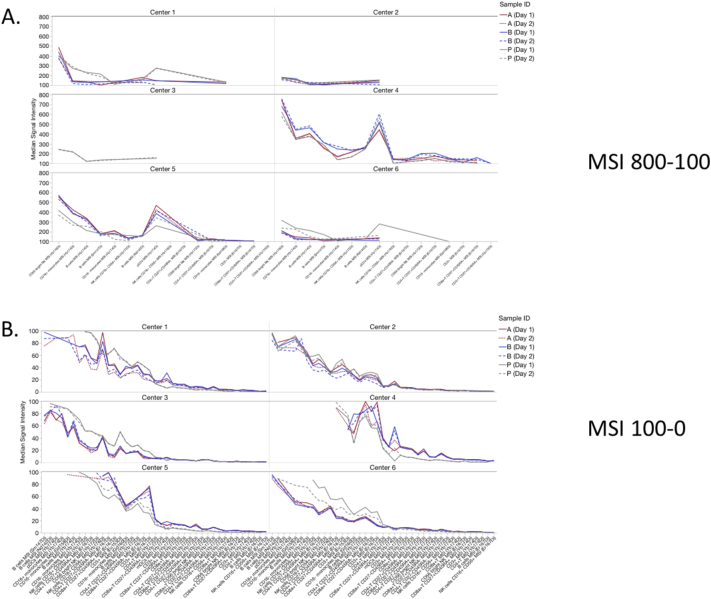
Center-by-center MSI of various manually-gated cell populations. Each line depicts a separate sample (A1, A2, B1, B2, P1, P2). Raw, unnormalized data are depicted. A. MSI ranging from 800 to 100 Dual Counts. B. MSI ranging below 100 Dual Counts.

For median signal intensities (MSI), populations with higher signal (> 100 Dual Counts) had smaller CVs, typically < 25% (Fig. 4A, Supp. Fig. S5). As the signal intensity decreased, the CVs increased, but usually remained < 50% (Fig. 4B). Taking all gated populations together, the average CV for each center was around 30% (Table 1). When pooling the data for all centers, the main effect of either normalization method was to increase the signal intensity across all populations, with little effect on average pooled CV (CV ~ 30%, Fig. 4, Table 1, Table S4). Center 2 and Center 3 often had the lowest MSI values but still acceptable resolution (Supp. Fig. S4, Supp. Fig. S5), while Center 4 and Center 5 typically had the highest MSI values.

As discussed above, the Fluidigm normalizer has a minimum bead requirement. If this is not met due to insufficient beads, the normalization program can fail. Three sites were affected by this (total of 9/36 files), including all six files from one site (Table 1). This was probably due to inadequate resuspension of the EQ bead aliquot supplied as part of the study reagents, leading to too few beads in the solution used to resuspend the cells during sample running. In case this would affect the comparison of the Fluidigm and MATLAB normalizers, the MATLAB normalizer was re-run on only those files that passed the Fluidigm normalizer, but this had no effect on the analysis outcome.

### 3.6 Intra- and inter-site differences-clustering algorithms

The manual gating analysis above enabled adjustments of the gates as necessary to account for staining differences between sites, as well as the increased background in certain channels for the site-stained samples. To explore to what extent this may affect automated clustering, principal component analysis (PCA) was first performed on the 36 MATLAB-all normalized files (Suppl. Fig. S7). Due to parameter-naming differences between certain sites, the FCS files first had to be harmonized using the cytofCore software package; Center_2_P1 was chosen as the template file. Since we wanted to investigate possible differences between sites, no attempt was made to batch-correct by site or any other way. Combining each of the six samples from each of the six centers and running a PCA for each sample highlighted the fact that no significant technical batch effect exists between centers. Similarly, calculating a PCA of all samples prepared by each center, highlights that samples are comparable in a high-dimensional setting.

Next, SPADE3 and Cytofkit (FlowSOM, ClusterX, and Rphenograph) analyses were performed on the same harmonized MATLAB-normalized files. The same 14 surface markers were used for clustering in both SPADE3 and Cytofkit (see Section 2.6). The files were analyzed in three ways: all 12 P files only, all 24 A and B files only (AB), and all 36 A, B, and P files together (ABP). This would potentially allow us to separate the differences from site-staining vs Prestained cells, from the differences inherent to each site's machine operation.

Regardless of the clustering algorithm used, the results were similar for the (Fig. S9, Fig. S10, Fig. S11, Fig. S12, Fig. S13, Fig. S14, Fig. S15, Fig. S16, Fig. S17, Fig. S18, Fig. S19, Fig. S20, Fig. S21, Fig. S22, Fig. S23, Fig. S24, Fig. S25, Fig. S26, Fig. S27, Fig. S28, Fig. S29, Fig. S30, Fig. S31, Fig. S32). SPADE3's Earthmover's Distance calculations between each site's individual samples showed that the P-only samples were more similar (EMD 0–2.4; Suppl. Fig. S12) than the AB-only samples (EMD 0–5.7; Suppl. Fig. S13) or all ABP samples (EMD 0–4.3; Suppl. Fig. S14). Based on the frequencies of each cluster in the ABP clustering calculations, each of the four algorithms demonstrated that the P samples from each site were similar to each other but distinct from the AB samples. Each algorithm also showed that the cluster frequencies were more similar for certain pairs of centers than other pairs. Centers 4 and 5 were paired, and Centers 1, 2, and 6 were a group; Center 3 remained an outlier in comparison.

## 4 Discussion

With the comparatively young age of mass cytometry, much standardization is still needed to address CyTOF variability over time and between instruments. Along with Tricot et al. (Tricot et al., 2015), this study represents one of the first tests of inter-site reproducibility of staining and sample acquisition. First, it is possible to generate basic Prestained control samples that are stable over time and tolerant of the rigors of shipping, though this needs to be tested on a case-by-case basis (Sumatoh et al., 2017). This approach allows the creation of an external standard sample to complement day-of staining controls in order to separate variation in experimental proficiency (cell thawing, stimulation, staining) from variation in instrument performance.

We focused this study as a test of the proficiency of each site to process samples and to acquire on their instruments. To remove site-specific variations in antibody conjugation efficiency, we used only commercially-available conjugated reagents. To avoid errors in preparation of the 20-antibody cocktail, the primary site generated one concentrated master mix and sent aliquots to each site. However, this revealed that the concentrated antibody cocktail was itself not stable and gave increased background for a handful of markers. Therefore, the ability to make concentrated cocktail ahead of time is strongly dependent on the exact antibodies used, and must be determined empirically. Since not all antibodies were affected, it may be possible to create stable mixes of fewer antibodies and only add in the more problematic ones on-site. Alternatively, lyophilization or other dry-down procedures may be utilized, but as yet, there are no mass cytometry publications to demonstrate how broadly this may apply.

Between the six sites, there were some errors in marker/channel naming that had to be resolved before batch analysis (manual or clustering) could occur. The new Helios mass cytometer (Fluidigm) has the ability to export a template file for experimental panels, which would resolve this issue in future studies.

All six sites gave similar results for manual gating, demonstrating overall technical proficiency. However, the concentration of beads added to the sample makes a significant difference in the ability to normalize samples; indeed, one site had all six files fail to pass Fluidigm normalization. Even without normalization, Frequency of Parent data are comparable (CV < 30%) between Prestained and site-stained samples. Similarly, MSI data are comparable (typical higher MSI CV < 25%; low MSI CV < 50%) between different days at the same site, and between different sites. Normalization made MSI data more comparable between sites.

Automated analysis using several different algorithms did detect slight differences between cluster frequencies between sites. As expected, the main difference was between the P samples and the combined AB samples, due in part to the increased background for certain antibodies used as clustering parameters. The fact that certain pairs of sites were more similar in cluster frequencies and Earthmover's distance than to other sites does suggest batch-correction would further facilitate removing the remaining inter-site differences.

Therefore, we conclude that inter-site studies are possible with mass cytometry to a level comparable to fluorescence flow cytometry (Finak et al., 2016, Kalina et al., 2015, Maecker et al., 2005).

The following are the supplementary data related to this article.

**Table S1:**
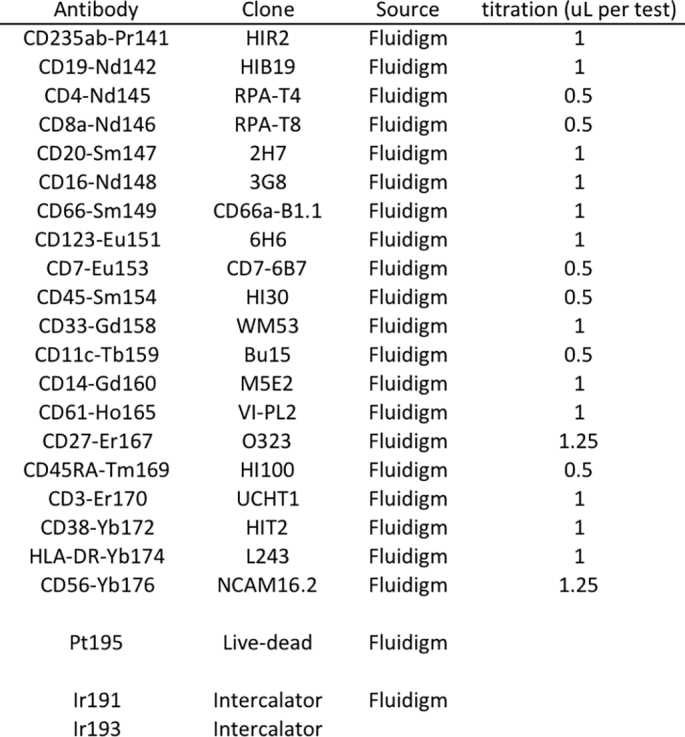
Antibody cocktail.

**Table S2:**
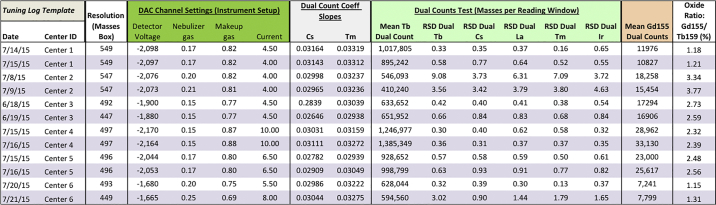
Tuning logs for each center for each day of samples.

Table S3 Summary statistics for CV of Frequency of Parent manually-gated populations. All centers, all populations, all samples. Table S3

Table S4 Summary statistics for CV of MSI for manually-gated populations. All centers, all populations, all samples. Table S4

**Fig. S6:**
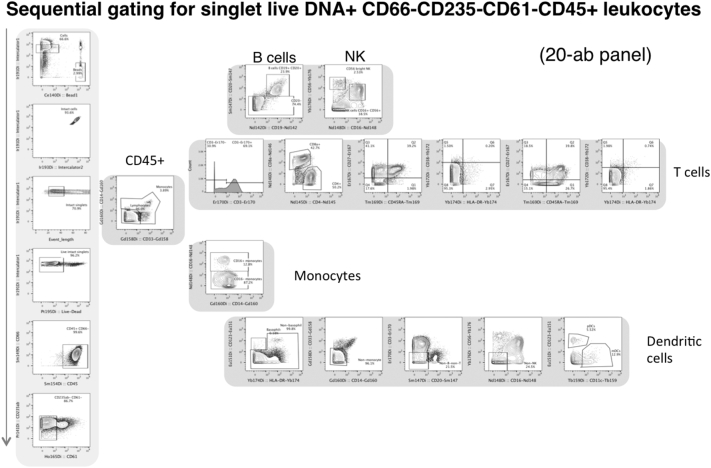
Gating strategy for multicenter experiment.

**Fig. S7:**
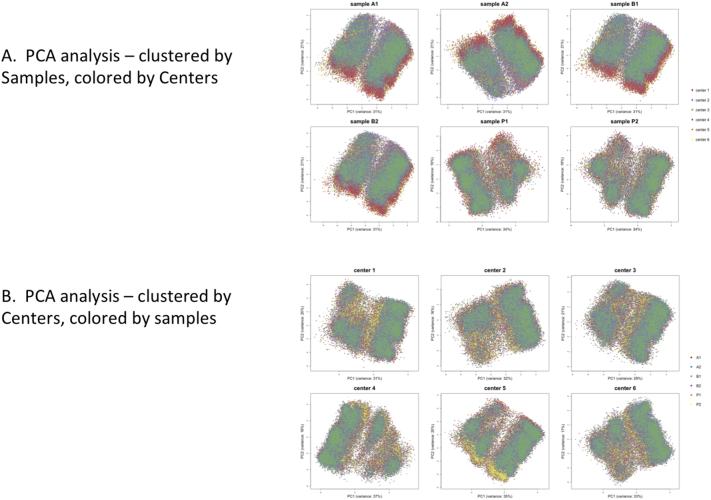
Principal component analysis – by center and by sample. All samples were included. A. PCA clustered by sample. The colors represent each of the six centers. B. PCA clustered by center. The colors represent each of the six samples.

**Fig. S8:**
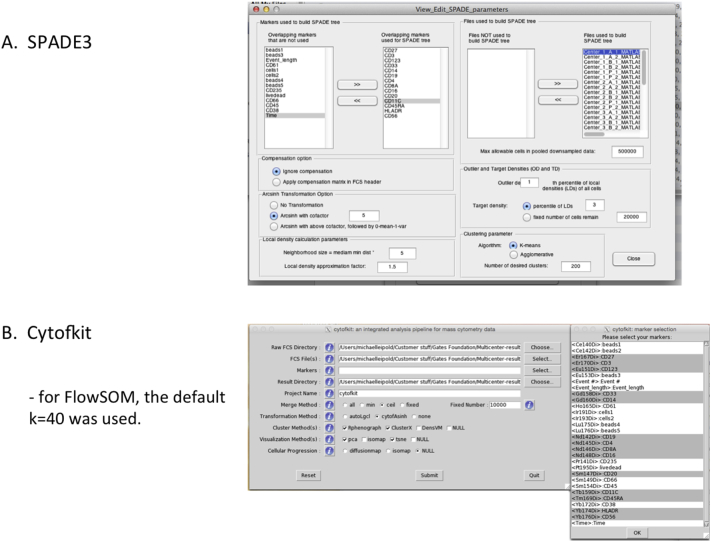
Screenshots of settings used for clustering algorithms. A. Settings used for MATLAB stand-alone SPADE3. B. Settings used for Cytofkit.

**Fig. S9:**
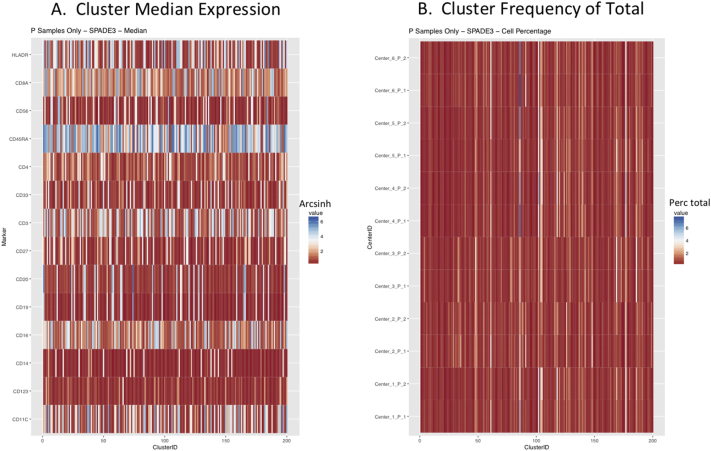
Heatmaps of SPADE3 results – P samples only – organized by Center. A. Cluster median expression (arcsinh). B. Cluster percent of total events.

**Fig. S10:**
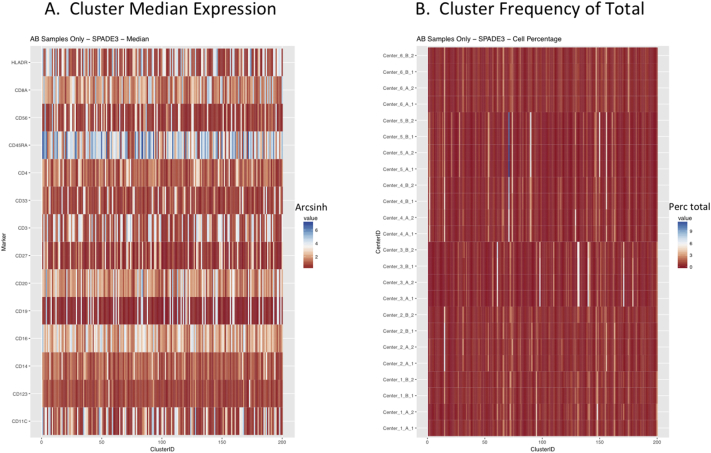
Heatmaps of SPADE3 results – AB samples only – organized by Center. A. Cluster median expression (arcsinh). B. Cluster percent of total events.

**Fig. S11:**
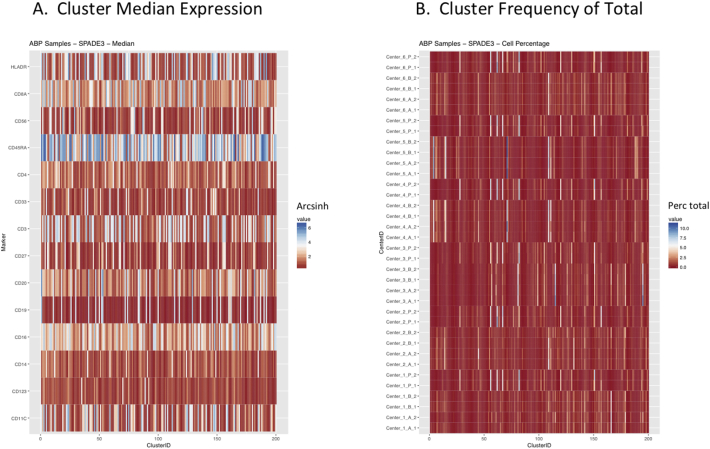
Heatmaps of SPADE3 results – all ABP samples – organized by Center. A. Cluster median expression (arcsinh). B. Cluster percent of total events.

**Fig. S12:**
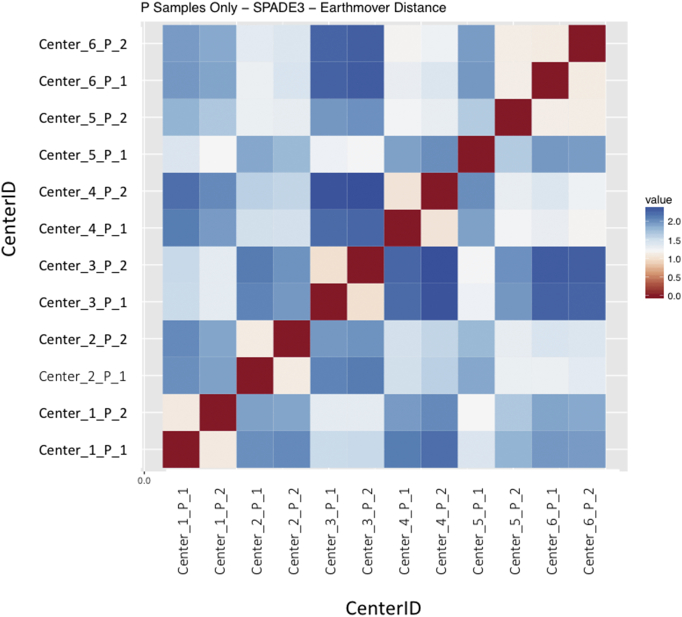
Heatmap of SPADE3 results – P samples only – Earthmover's distance between samples – organized by Center.

**Fig. S13:**
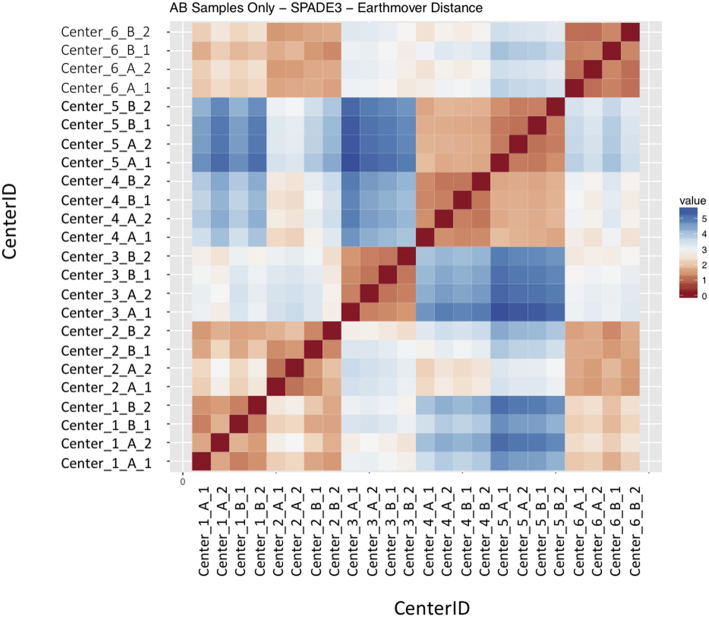
Heatmap of SPADE3 Results – AB samples only – Earthmover's distance between samples – organized by Center.

**Fig. S14:**
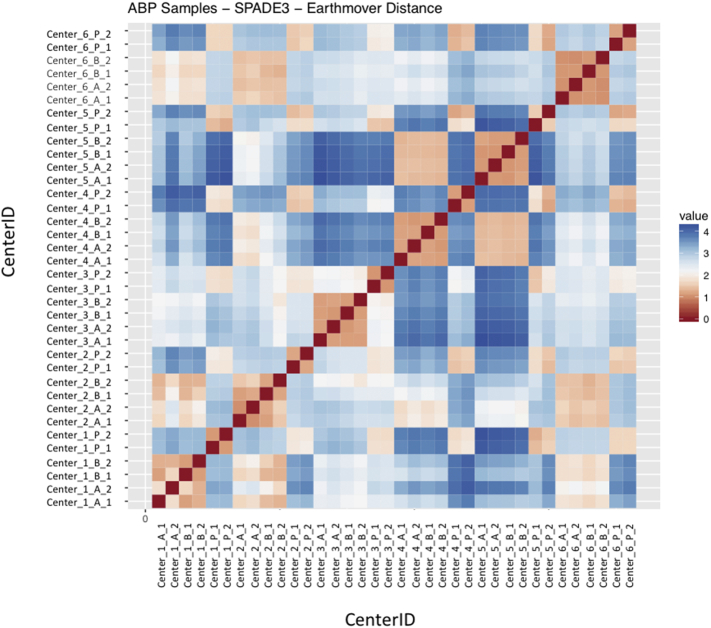
Heatmap of SPADE3 Results – All ABP samples – Earthmover's distance between samples – organized by Center.

**Fig. S15:**
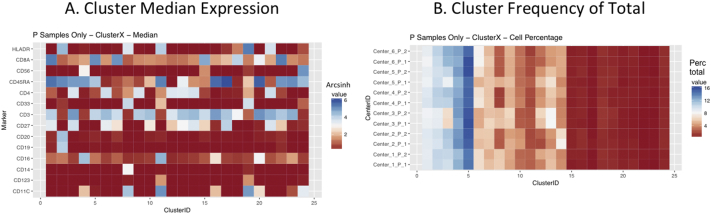
Heatmap of Cytofkit - ClusterX results – P samples only – organized by Center. A. Cluster median expression (arcsinh). B. Cluster percent of total events.

**Fig. S16:**
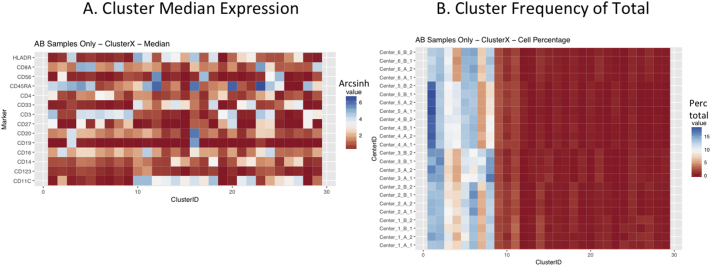
Heatmap of Cytofkit - ClusterX results – AB samples only – organized by Center. A. Cluster median expression (arcsinh). B. Cluster percent of total events.

**Fig. S17:**
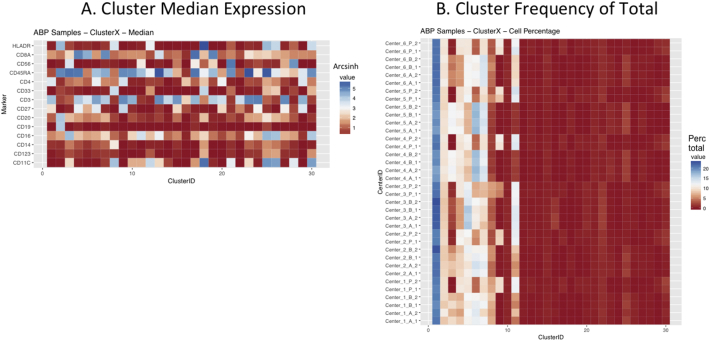
Heatmap of Cytofkit - ClusterX results – All ABP samples – organized by Center. A. Cluster median expression (arcsinh). B. Cluster percent of total events.

**Fig. S18:**
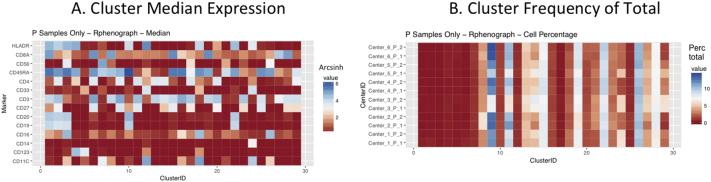
Heatmap of Cytofkit - phenograph results – P samples only – organized by center. A. Cluster median expression (arcsinh). B. Cluster percent of total events.

**Fig. S19:**
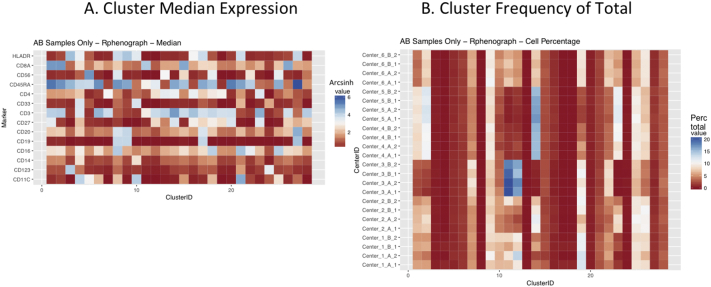
Heatmap of Cytofkit - phenograph results – AB samples only – organized by center. A. Cluster median expression (arcsinh). B. Cluster percent of total events.

**Fig. S20:**
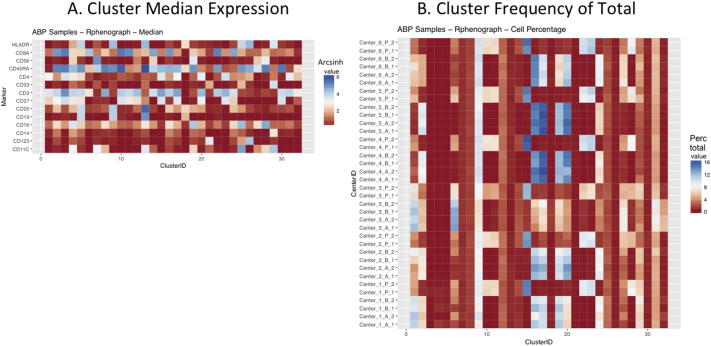
Heatmap of Cytofkit - phenograph results – All ABP samples – organized by center. A. Cluster median expression (arcsinh). B. Cluster percent of total events.

**Fig. S21:**
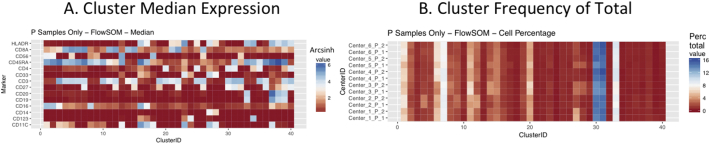
Heatmap of Cytofkit - FlowSOM results – P samples only – organized by center. A. Cluster median expression (arcsinh). B. Cluster percent of total events.

**Fig. S22:**
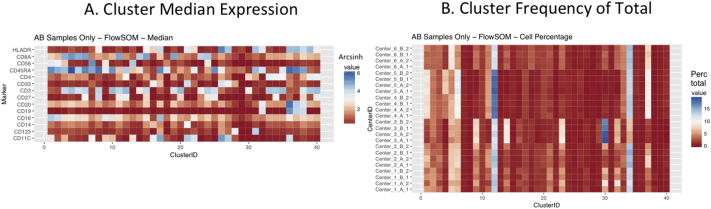
Heatmap of Cytofkit - FlowSOM results – AB samples only – organized by center. A. Cluster median expression (arcsinh). B. Cluster percent of total events.

**Fig. S23:**
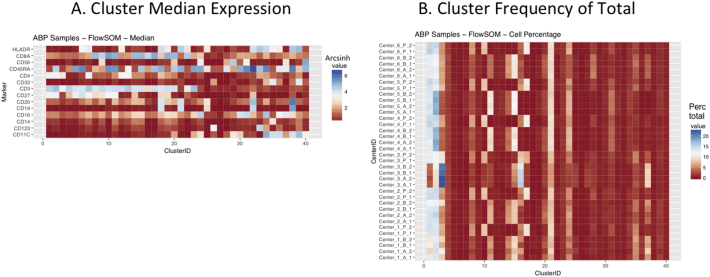
Heatmap of Cytofkit - phenograph results – All ABP samples – organized by Center. A. Cluster median expression (arcsinh). B. Cluster percent of total events.

**Fig. S24:**
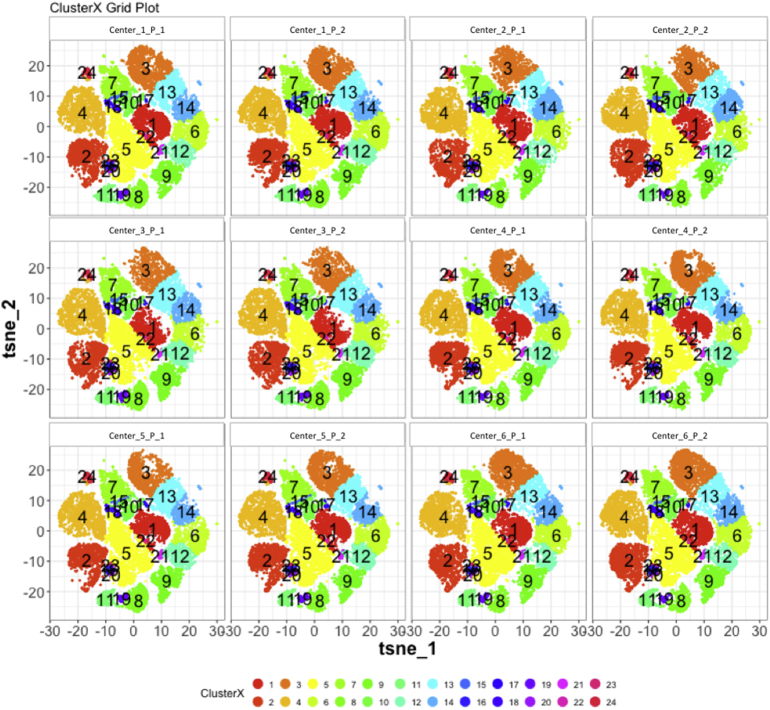
tSNE plots from Cytofkit - ClusterX results - P samples only. Arranged by sample.

**Fig. S25:**
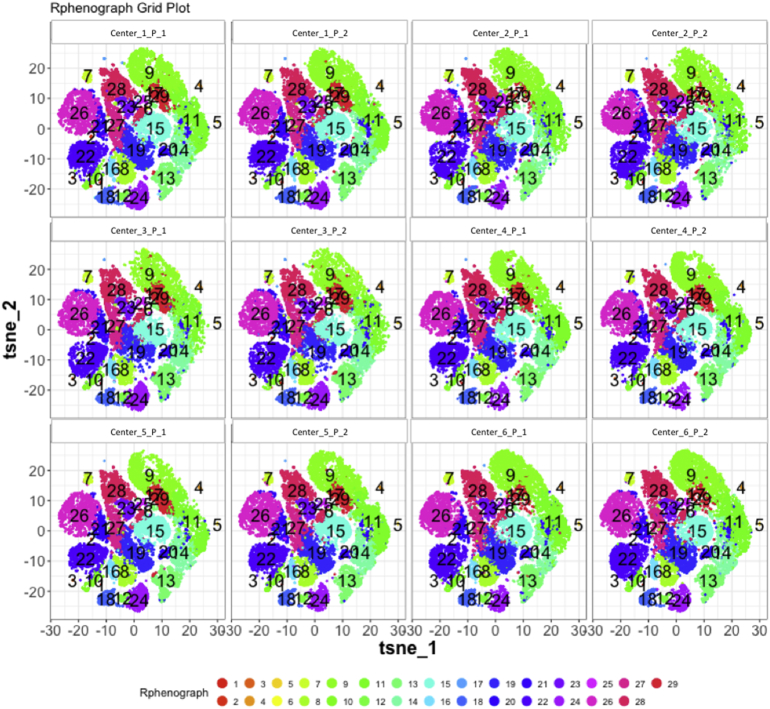
tSNE plots from Cytofkit - phenograph results - P samples only. Arranged by sample.

**Fig. S26:**
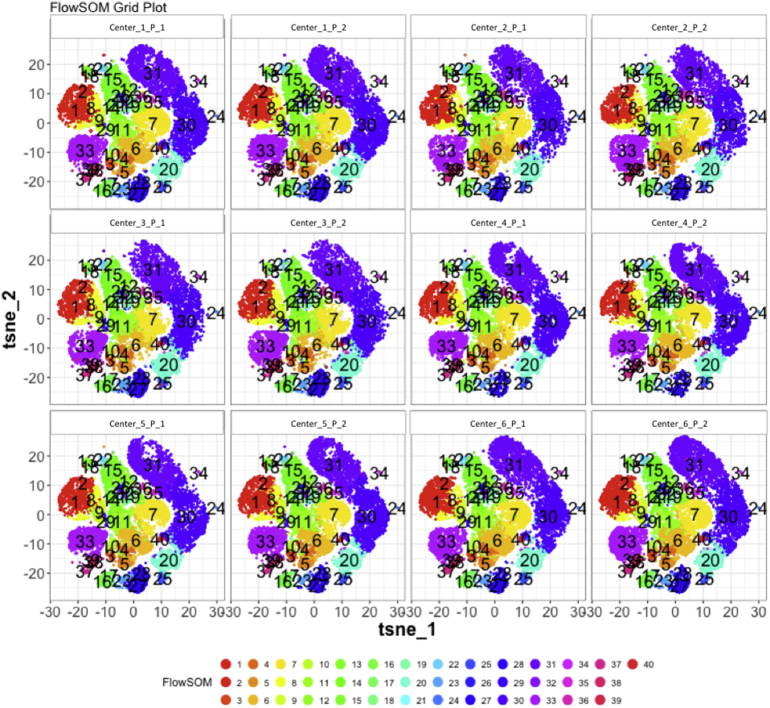
tSNE plots from Cytofkit - FlowSOM results - P samples only. Arranged by sample.

**Fig. S27:**
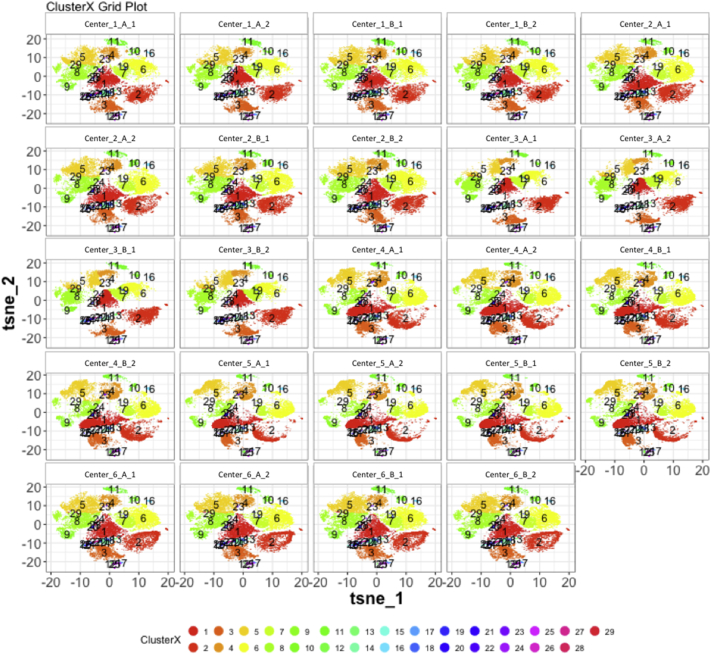
tSNE plots from Cytofkit - ClusterX results - AB samples only. Arranged by sample.

**Fig. S28:**
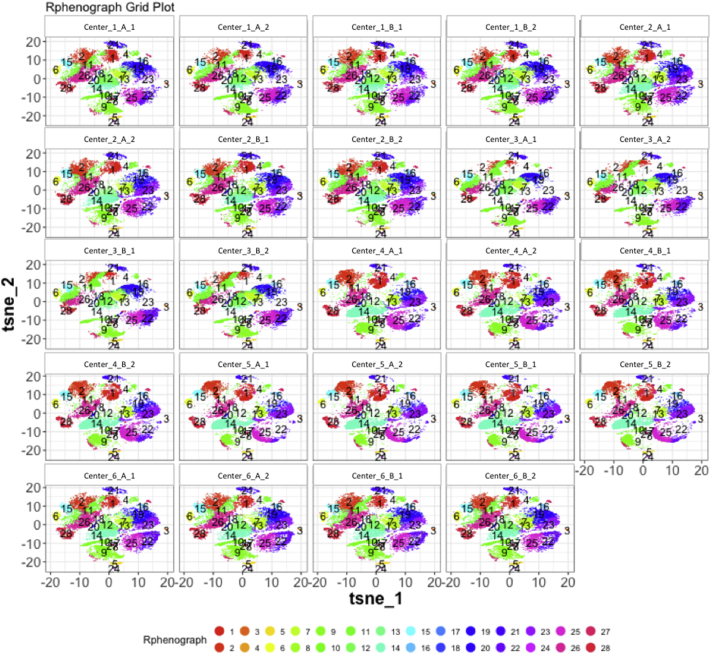
tSNE plots from Cytofkit - phenograph results - AB samples only. Arranged by sample.

**Fig. S29:**
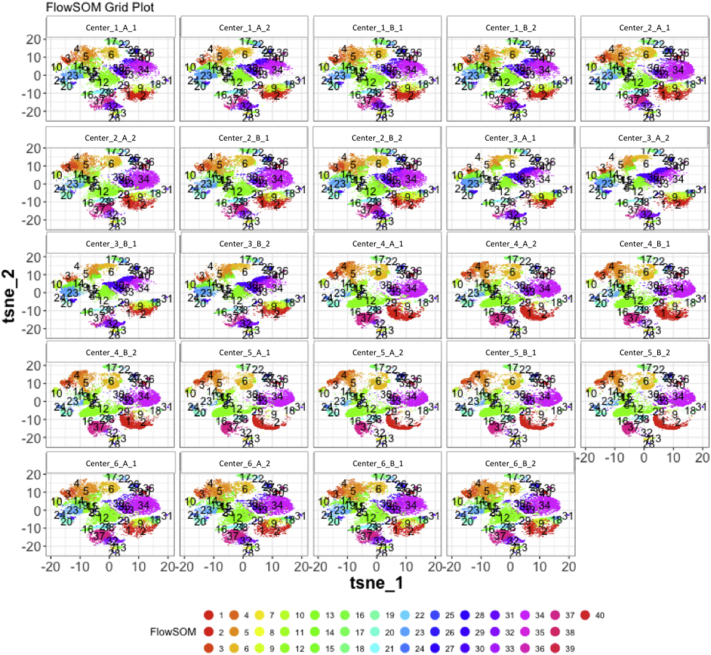
tSNE plots from Cytofkit - FlowSOM results - AB samples only. Arranged by sample.

**Fig. S30:**
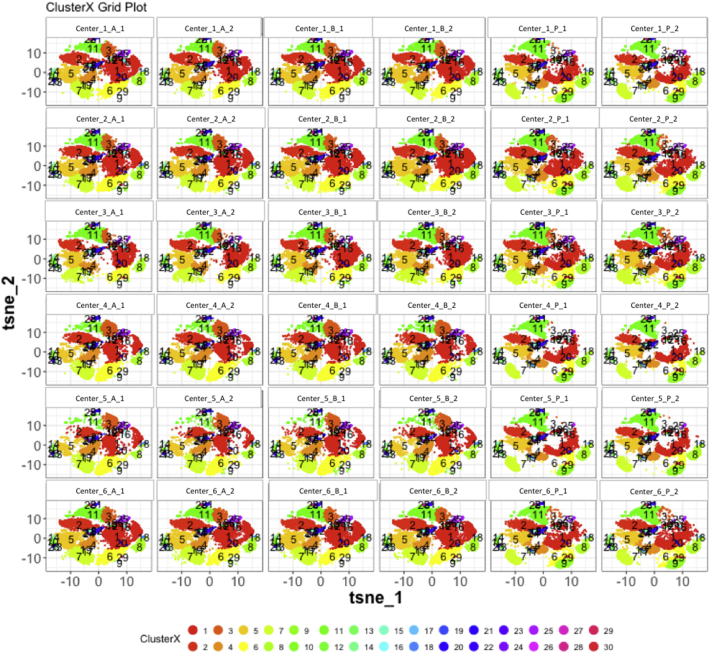
tSNE plots from Cytofkit - ClusterX results - All ABP samples only. Arranged by sample.

**Fig. S31:**
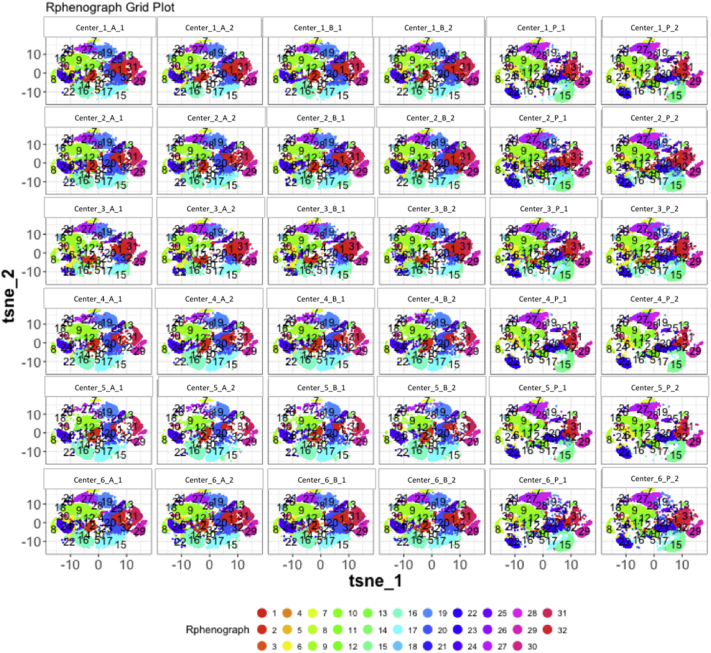
tSNE plots from Cytofkit - phenograph results - All ABP samples only. Arranged by sample.

**Fig. S32:**
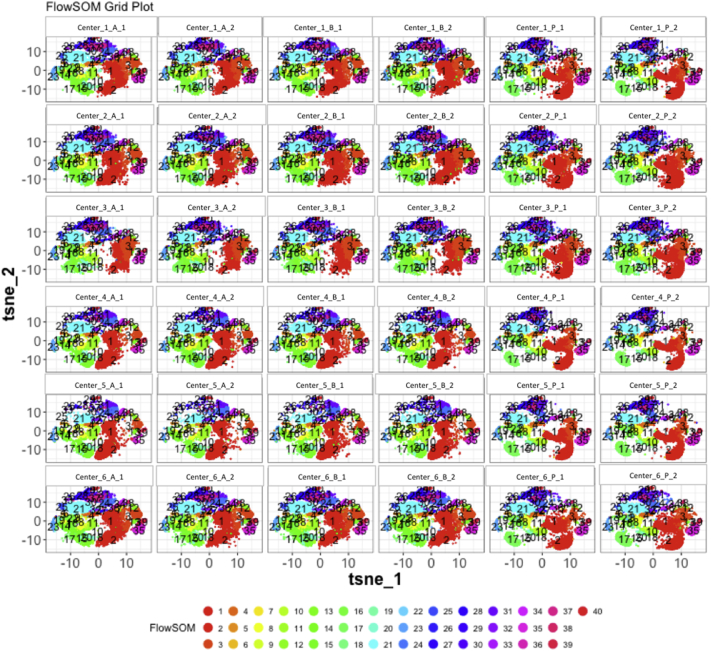
tSNE plots from Cytofkit - FlowSOM results - All ABP samples only. Arranged by sample.
